# Serum 8-hydroxy-2-deoxyguanosine levels in pediatric acute lymphoblastic leukemia: A retrospective case–control study

**DOI:** 10.1097/MD.0000000000050247

**Published:** 2026-08-14

**Authors:** Meriban Karadoğan, Fatma Turkan Polat, Veysel Gok, Derya Koçer, Veysel Uzen

**Affiliations:** a Department of Pediatric Hematology and Oncology, Kayseri City Hospital, Kayseri, Turkey; b Department of Pediatric Hematology and Oncology, Erciyes University, Kayseri, Turkey; c Department of Clinical Biochemistry, Kayseri City Hospital, Kayseri, Turkey.

**Keywords:** 8-OHdG, acute lymphoblastic leukemia, biomarker, oxidative stress, pediatric

## Abstract

Oxidative stress contributes to carcinogenesis through deoxyribonucleic acid (DNA) damage, and 8-hydroxy-2-deoxyguanosine (8-OHdG) is a well-established biomarker of oxidative DNA injury. This study aimed to evaluate oxidative stress in pediatric acute lymphoblastic leukemia (ALL) by measuring serum 8-OHdG levels before and after chemotherapy. This retrospective case–control study included 32 children diagnosed with ALL and 30 age- and sex-matched healthy controls. Serum 8-OHdG levels were measured using an enzyme-linked immunosorbent assay prior to treatment and after the initiation of maintenance therapy, approximately one month following intensive chemotherapy. Comparisons between groups were performed using the Mann–Whitney *U* test, and within-patient changes were assessed using the Wilcoxon signed-rank test. Pretreatment serum 8-OHdG levels were significantly higher in children with ALL compared with controls (*P* < .001), indicating increased oxidative DNA damage. Serum 8-OHdG levels significantly decreased after chemotherapy in children with ALL (*P* < .001). However, no statistically significant difference was observed between post-treatment 8-OHdG levels and those of the control group (*P* = 1.0). No significant associations were found between pretreatment 8-OHdG levels and white blood cell count or body mass index. Children with ALL exhibit significantly elevated serum 8-OHdG levels, supporting a role for oxidative stress in disease pathogenesis. The reduction in 8-OHdG levels following chemotherapy suggests a potential relationship between treatment response and oxidative DNA damage, indicating that 8-OHdG may serve as a useful biomarker in pediatric ALL.

## 1. Introduction

Acute lymphoblastic leukemia (ALL) is the most common malignancy in childhood; however, its etiopathogenesis has not yet been fully elucidated.^[1]^ Increasing evidence suggests that oxidative stress plays a critical role in carcinogenesis through both genetic and epigenetic mechanisms. Oxidative stress can induce lipid peroxidation, protein misfolding, deoxyribonucleic acid (DNA) damage, and mitochondrial dysfunction, all of which may contribute to genomic instability, altered cellular signaling, and apoptosis.^[2,3]^

A widely used biomarker for assessing oxidative DNA damage is 8-hydroxy-2-deoxyguanosine (8-OHdG), a stable product of oxidative modification of guanine bases in DNA.^[4,5]^ Elevated levels of 8-OHdG have been reported in various malignancies and are considered indicative of increased oxidative stress. In pediatric ALL, both the disease itself and multi-agent chemotherapy regimens – such as vincristine, cytosine arabinoside, and methotrexate – may enhance the production of reactive oxygen species, thereby increasing oxidative stress. Chemotherapy-related oxidative damage has also been associated with acute and long-term toxicities, including neurotoxicity and neurocognitive impairment in children.^[6,7]^

Despite growing interest in oxidative stress in leukemia, most previous studies have focused on urinary 8-OHdG levels. Data on serum 8-OHdG levels in pediatric ALL, particularly regarding longitudinal changes during treatment, remain limited. Furthermore, the relationship between serum 8-OHdG levels and clinical characteristics in this population has not been fully clarified.

Therefore, this study aimed to evaluate serum 8-OHdG levels in children with ALL before and after chemotherapy and to compare these levels with those of healthy controls. We also investigated the relationship between serum 8-OHdG levels and clinical parameters such as age, sex, white blood cell (WBC) count, and body mass index (BMI).

We hypothesized that pretreatment serum 8-OHdG levels would be higher in pediatric ALL patients than in healthy controls and would decrease following chemotherapy.

## 2. Material and methods

### 2.1. Study design and setting

This study was designed as a retrospective case–control study and was conducted at Kayseri City Hospital, Turkey. Clinical and laboratory data were retrospectively retrieved from existing medical records. Serum samples had been collected previously and stored under appropriate conditions. Following ethics committee approval, these archived serum samples were used in the present secondary analysis to measure serum 8-hydroxy-2-deoxyguanosine (8-OHdG). No additional patient enrollment or prospective sample collection was undertaken for this study.

### 2.2. Participants

The case group consisted of 32 pediatric patients diagnosed with ALL (13 males and 19 females) who had not received any treatment prior to diagnosis. The median age of the patients was 7.1 years.

The control group included 30 age- and sex-matched healthy children selected from those undergoing routine physical examinations at the same hospital during the same period. Children who had used antioxidant or multivitamin supplements within 4 weeks prior to blood sampling were excluded from both groups.

### 2.3. Diagnosis and treatment protocol

The diagnosis of ALL was established using standard morphological, immunophenotypic, and cytogenetic criteria. All patients were treated according to the ALLIC-BFM 2019 treatment protocol. Demographic and clinical characteristics were obtained from hospital records.

### 2.4. Sample collection and group stratification

Archived serum samples that had been collected previously were used for the present secondary analysis. Serum 8-hydroxy-2-deoxyguanosine (8-OHdG) levels were measured in ALL patients at 2 time points: before treatment initiation and after the initiation of the maintenance phase, approximately one month following intensive chemotherapy. Serum 8-OHdG levels were also measured in the control group for comparison.

Pretreatment stratification of ALL patients was performed based on WBC count (<20,000/mL or ≥20,000/mL) and BMI. BMI values at or above the 85th percentile according to pediatric growth charts were classified as overweight or obese.

### 2.5. Measurement of serum 8-OHdG

Previously collected serum samples, which had been stored at −80°C under appropriate conditions, were used for the present secondary analysis. Serum 8-hydroxy-2-deoxyguanosine (8-OHdG) concentrations were measured using a commercially available enzyme-linked immunosorbent assay kit (USCN, Cloud-Clone Corp., Hubei, China) with a detection range of 74 to 6000 pg/mL, according to the manufacturer’s instructions. All archived serum samples were analyzed under identical laboratory conditions. Sample concentrations were calculated using standard calibration curves.

### 2.6. Statistical analysis

Statistical analyses were performed using Statistical Package for the Social Sciences software. Data distribution was assessed for normality. Continuous variables were summarized as median and interquartile range. Comparisons between independent groups were conducted using the Mann–Whitney *U* test, while within-group comparisons were performed using the Wilcoxon signed-rank test. Categorical variables were analyzed using the chi-square test. Subgroup analyses were performed according to WBC count and BMI categories. Only participants with complete clinical and laboratory data were included in the analyses; therefore, no imputation for missing data was required. No sensitivity analyses were conducted due to the retrospective design and limited sample size.

### 2.7. Ethical approval

This study was conducted in accordance with the ethical principles of the Declaration of Helsinki and was approved by the Ethics Committee of Kayseri City Hospital (date: May 18, 2022; approval number: 641). Written informed consent was obtained from the parents or legal guardians of all participants prior to inclusion in the study.

## 3. Results

A total of 32 pediatric patients with ALL and 30 healthy controls were included in the analysis. The mean age of the control group was 9.4 ± 4.4 years (median, 9 years), whereas the mean age of the ALL patients was 8.3 ± 5.7 years (median, 7.1 years). Males constituted 56.3% (n = 18) of the control group and 53.1% (n = 17) of the ALL group. There were no statistically significant differences between the case and control groups with respect to age or sex.

Pretreatment serum 8-hydroxy-2-deoxyguanosine (8-OHdG) levels (pg/mL) were significantly higher in the ALL patients compared with the healthy controls (Table 1, *P* < .001).

**Table 1:** Comparison of pretreatment serum 8-OHdG levels between ALL patients and healthy controls.

	Control (n = 30)	Patient (n = 32)	*P*
Age, median (IQR)	8.5 (5–12.3)	5.1 (3.1–13.7)	.367*
Age, n (%)			
<10 yr	17 (56.7)	18 (56.3)	1.0†
≥10 yr	13 (43.3)	14 (43.8)	
Gender, n (%)			
Female	15 (50)	14 (43.8)	.812†
Male	15 (50)	18 (56.3)	
8-OHdG (before treatment), median (IQR)	369.8 (331.4–494.1)	851 (658.5–1101.8)	<.001*
8-OHdG (after treatment), median (IQR)	369.8 (331.4–494.1)	413.7 (309.8–485.3)	1.0*

8-OHdG = 8-hydroxy-2-deoxyguanosine, ALL = acute lymphoblastic leukemia, IQR = interquartile range (25th–75th percentile).

There were no significant differences (*P* > .05) in the age and sex of the patients between the case and control groups. Pretreatment serum 8-OHdG levels were significantly higher in the ALL group compared to controls (*P* < .001).

* Mann–Whitney *U* test.

† Chi-square test.

After chemotherapy, serum 8-OHdG levels (pg/mL) in the ALL group decreased significantly compared with pretreatment levels (Table 2, *P* < .001). However, no statistically significant difference was observed between post-treatment 8-OHdG levels and those of the control group (Table 1, *P* = 1.0).

**Table 2:** Changes in serum 8-OHdG levels in ALL patients before and after chemotherapy.

	Before	After	Variation (%)	*P* *
8-OHdG, median (IQR)	851 (658.5–1101.8)	413.7 (309.8–485.3)	−54.6 (−67.3 to −43.1)	<.001

The post-treatment 8-OHdG levels in the ALL group showed a significant decrease compared to the pretreatment levels (*P* < .001).

8-OHdG = 8-hydroxy-2-deoxyguanosine, ALL = acute lymphoblastic leukemia, IQR = interquartile range (25th–75th percentile).

* Wilcoxon signed rank test.

The distribution of ALL subtypes, risk group classifications, and median WBC counts are presented in Table 3.

**Table 3:** Acute lymphoblastic leukemia (ALL) subtypes, risk groups, and median WBC counts.

Type of leukemia	n (%)
B ALL	29 (90.6)
T ALL	3 (9.4)
Risk group	n (%)
SR	9 (28.1)
MR	21 (65.6)
HR	2 (6.3)
WBC/µL, median (IQR)	7795 (3617.5–43,082.5)

HR = high risk, IQR = interquartile range, MR = medium risk, SR = standard risk, WBC = white blood cell.

When pretreatment serum 8-OHdG levels (pg/mL) were stratified according to WBC count (<20,000/µL vs ≥20,000/µL) and BMI (normal vs overweight/obese), no statistically significant differences were observed (Table 4, *P* > .05 for all comparisons).

**Table 4:** Comparison of pretreatment serum 8-OHdG levels by white blood cell count and weight status: ALL patients.

		8-OHdG, median (IQR)
WBC	n (%)	
<20,000/µL	21 (65.6)	874.2 (666.4–1119.8)
≥20,000/µL	11 (34.4)	768.5 (605.1–1060.4)
*P* *		.639
Overweight	n (%)	
No	21 (65.6)	854.8 (613.1–1094.6)
Yes	11 (34.4)	847.1 (741.5–1149.7)
*P* *		.815

No significant differences were observed in pretreatment 8-OHdG levels according to WBC categories (<20,000/µL vs ≥20,000/µL) or weight status (*P* > .05).

8-OHdG = 8-hydroxy-2-deoxyguanosine, ALL = acute lymphoblastic leukemia, IQR = interquartile range (25th–75th percentile), WBC = white blood cell.

* Mann–Whitney *U* test.

## 4. Discussion

### 4.1. Key findings

In the present study, children with ALL exhibited significantly higher pretreatment serum 8-hydroxy-2-deoxyguanosine (8-OHdG) levels compared with healthy controls, indicating increased oxidative DNA damage. These findings are consistent with current knowledge that oxidative stress contributes to carcinogenesis through DNA damage and related molecular mechanisms.^[1–4]^ Following chemotherapy, serum 8-OHdG levels decreased significantly; however, no statistically significant difference was observed between post-treatment levels and those of the control group, suggesting a partial normalization of oxidative stress after treatment.

### 4.2. Comparison with previous studies

Elevated levels of oxidative DNA damage in pediatric leukemia have been reported previously. Yang et al demonstrated significantly higher urinary 8-OHdG levels in Chinese children with acute leukemia prior to treatment compared with healthy controls, particularly in younger patients.^[8]^ Similarly, Sentürker et al reported increased oxidative DNA base damage and altered antioxidant enzyme levels in lymphocytes of children with ALL, supporting enhanced free radical activity in malignant cells.^[9]^

Dynamic changes in oxidative DNA damage during chemotherapy have also been described. Kennedy et al observed decreased 8-OHdG levels following the initiation of chemotherapy, with subsequent increases during intensive treatment phases.^[10]^ In addition, declining antioxidant status during chemotherapy has been associated with increased treatment-related toxicity and poorer clinical outcomes in pediatric ALL patients.^[11]^ These observations are consistent with the reduction in serum 8-OHdG levels observed after chemotherapy in the present study.

### 4.3. Biological and clinical implications

Oxidative stress has also been implicated in treatment-related neurotoxicity. Dewan et al reported higher cerebrospinal fluid 8-OHdG levels in children with chemotherapy-induced neurotoxicity, suggesting increased oxidative damage within the central nervous system during treatment.^[12]^

Beyond pediatric leukemia, elevated urinary 8-OHdG levels have been reported in several adult malignancies and chronic diseases, including prostate cancer, breast cancer, lung cancer, diabetes, and atherosclerosis, highlighting the role of 8-OHdG as a general biomarker of oxidative DNA damage.^[13–18]^ In patients with hematological malignancies, particularly adult T-cell leukemia/lymphoma, markedly increased urinary 8-OHdG levels have been observed.^[18]^

Chemotherapeutic agents commonly used in the treatment of ALL, including vincristine, anthracyclines, and alkylating agents, are known to induce cytotoxicity partly through the generation of reactive oxygen species.^[19–21]^

Recent studies have further demonstrated that oxidative stress levels may vary during different phases of leukemia treatment. Elevated oxidative stress during intensive chemotherapy and persistent oxidative damage during maintenance therapy have been reported in pediatric ALL patients.^[22,23]^ Moreover, chemotherapy- and radiation-induced oxidative stress may persist for prolonged periods, potentially contributing to late tissue injury and long-term complications.^[24–26]^

Importantly, while urinary 8-OHdG reflects cumulative excretion of oxidative DNA damage products, serum 8-OHdG may better represent immediate and dynamic changes in oxidative stress, particularly during different phases of chemotherapy.

### 4.4. Limitations

Several limitations of this study should be acknowledged. The retrospective secondary analysis of archived serum samples, the single-center design, and the relatively small sample size may limit statistical power and the precision of effect estimates. Additionally, only serum 8-OHdG levels were measured, whereas oxidative stress may differ across biological compartments. Measurement of 8-OHdG in urine or cerebrospinal fluid may provide further insight into systemic versus localized oxidative stress.^[8,12,18]^ Furthermore, potential confounding factors such as dietary habits, genetic susceptibility, and environmental exposures were not evaluated. These limitations may have influenced the magnitude of the observed associations but are unlikely to affect their overall direction.

### 4.5. Generalisability

The findings of this study may be generalizable to pediatric patients with ALL treated in similar clinical settings using comparable chemotherapy protocols. However, extrapolation to other populations, treatment regimens, or healthcare systems should be undertaken with caution.

## 5. Conclusions

In conclusion, pediatric patients with ALL demonstrate increased oxidative DNA damage, as reflected by elevated serum 8-OHdG levels, particularly before treatment. The observed reduction following chemotherapy suggests a potential association between oxidative stress and treatment response. Given the established role of oxidative stress in leukemogenesis and treatment-related toxicity, further prospective studies with larger sample sizes and comprehensive oxidative stress profiling are warranted to clarify the clinical utility of 8-OHdG as a biomarker and to explore targeted oxidative stress–modulating strategies in pediatric ALL.

## Author contributions

**Conceptualization:** Meriban Karadoğan.

**Data curation:** Meriban Karadoğan, Derya Koçer.

**Formal analysis:** Fatma Turkan Polat.

**Funding acquisition:** Meriban Karadoğan.

**Investigation:** Meriban Karadoğan, Derya Koçer.

**Methodology:** Meriban Karadoğan, Veysel Gok, Derya Koçer.

**Project administration:** Veysel Uzen.

**Resources:** Fatma Turkan Polat, Veysel Uzen.

**Software:** Derya Koçer, Veysel Uzen.

**Supervision:** Meriban Karadoğan, Veysel Uzen.

**Validation:** Fatma Turkan Polat, Veysel Gok, Derya Koçer.

**Visualization:** Meriban Karadoğan, Veysel Gok.

**Writing – original draft:** Meriban Karadoğan, Veysel Gok.

**Writing – review & editing:** Meriban Karadoğan.
